# Zinc Oxide Nanocomposites—Extracellular Synthesis, Physicochemical Characterization and Antibacterial Potential

**DOI:** 10.3390/ma13194347

**Published:** 2020-09-30

**Authors:** Paweł Pomastowski, Anna Król-Górniak, Viorica Railean-Plugaru, Bogusław Buszewski

**Affiliations:** 1 Centre for Modern Interdisciplinary Technologies, Nicolaus Copernicus University in Torun, 4 Wileńska Str., 87-100 Torun, Poland; annkrol18@gmail.com (A.K.-G.); viorica.railean@umk.pl (V.R.-P.); bbusz@chem.umk.pl (B.B.); 2 Department of Environmental Chemistry and Bioanalytics, Faculty of Chemistry, Nicolaus Copernicus University in Torun, 7 Gagarina Str., 87-100 Torun, Poland

**Keywords:** zinc oxide, nanocomposites, extracellular synthesis, *Lactobacillus paracasei*, organic deposit, antimicrobial activity, mechanism of formation

## Abstract

This research presents, for the first time, the potential of the *Lactobacillus paracasei* LC20 isolated from sweet whey as a novel, effective and accessible source for post-cultured ZnO nanocomposites synthesis. The obtained nanocomposites were subjected to comprehensive characterization by a broad spectrum of instrumental techniques. Results of spectroscopic and microscopic analysis confirmed the hexagonal crystalline structure of ZnO in the nanometer size. The dispersion stability of the obtained nanocomposites was determined based on the zeta potential (ZP) measurements—the average ZP value was found to be −29.15 ± 1.05 mV in the 7–9 pH range. The ZnO nanocomposites (NCs) demonstrated thermal stability up to 130 °C based on the results of thermogravimetric TGA/DTG) analysis. The organic deposit on the nanoparticle surface was recorded by spectroscopic analysis in the infrared range (FT-IR). Results of the spectrometric study exhibited nanostructure-assisted laser desorption/ionization effects and also pointed out the presence of organic deposits and, what is more, allowed us to identify the specific amino acids and peptides present on the ZnO NCs surfaces. In this context, mass spectrometry (MS) data confirmed the nano-ZnO formation mechanism. Moreover, fluorescence data showed an increase in fluorescence signal in the presence of nanocomposites designed for potential use as, e.g., biosensors. Despite ZnO NCs’ luminescent properties, they can also act as promising antiseptic agents against clinically relevant pathogens. Therefore, a pilot study on the antibacterial activity of biologically synthesized ZnO NCs was carried out against four strains (*Escherichia coli, Staphylococcus aureus, Klebsiella pneumoniae* and *Pseudomonas aeruginosa*) by using MIC (minimal inhibitory concentration). Additionally, the colony forming units (CFU) assay was performed and quantified for all bacterial cells as the percentage of viable cells in comparison to a control sample (untreated culture) The nanocomposites were effective among three pathogens with MIC values in the range of 86.25–172.5 μg/mL and showed potential as a new type of, e.g., medical path or ointment formulation.

## 1. Introduction

The production of metal oxide nanomaterials such as zinc oxide nanocomposites (ZnO NCs) is an emerging and currently researched subject in nanotechnology [1,2]. Over the years, synthesis of nanomaterials has been of research interest but there is a growing need for development of more efficient, facile and environmentally friendly methods. Therefore, synthesis of nano-ZnO using biological systems is attracting rising attention, mainly due to the lower consumption of hazardous reagents and lower cytotoxicity of the obtained nanomaterials (in comparison with traditional chemical and physical methods). Biosynthesis of zinc oxide can be carried out with various biological materials including bacteria, fungi and plant extracts [3,4,5]. There is limited evidence of effective ZnO nanoparticle formation using plants, whereas the adoption of a microbiological approach for this purpose has still not been sufficiently described. Among methods of microbial synthesis, two main types can be identified—intra- and extracellular methods. Intracellular production involves bacteria biomass for the nanocomposite formation, while the extracellular approach, known also as the post-cultured method, excludes microbial cells and uses a supernatant rich in biologically active compounds (e.g., enzymes or metabolites) [6]. As has been highlighted by literature data [7,8,9], the post-cultured method (extracellular synthesis) has its advantages, such as lower cost, simpler downstream processing (e.g., nanomaterial separation and purification processes) and possibility to reuse the bacterial cultures. Moreover, the extracellular approach is also a more adequate choice to produce nanocomposites with organic deposits on their surfaces coming from microbial compounds of natural origin. Railean-Plugaru et al. [10] obtained bio-AgNCs with the specific organic deposit connected with the silver core of nanoparticles. According to the latest literature data, there is no paper describing ZnO NCs synthesized by lactic acid bacteria (LAB) strain, naturally coated by organic deposit.

Despite the type of nanomaterial synthesis, it is necessary to consider the parameters such as selection of the organisms, as well as the precursor salts, that are the most suitable for this purpose and the type of synthesis. Kalpana et al. [4] used cell filtrates of *Aspergillus niger* to perform ZnO NP production. The nanomaterial obtained in this work revealed antimicrobial activity and showed the potential for dye degradation. Another example is the work of Kundu et al. [11], who performed the synthesis of ZnO nanoparticles by using cell-free filtrate after *Rhodococcus pyridinivorans* NT2 culturing and zinc sulfate as a precursor. They underlined the critical role of extracellular bioactive compounds of the strain (e.g., proteins) in the formation and stabilization of nano-ZnO.

LABs exhibit beneficial effects on humans—they colonize the digestive system involved in normal digestive processes (microflora) or act as a protective barrier on the skin’s surface. Moreover, their metabolites are generally regarded as safe and exhibit antimicrobial activity [12,13]. However, there are only a few reports describing the efficient and intracellular synthesis of nano-ZnO by using lactic acid bacteria biomass [14,15,16]. The lack of papers related to the post-cultured ZnO NP formation method with LAB metabolites has forced scientists to develop a novel, efficient and simple synthesis protocol. Designing metal nanocomposites based on biologically active and accessible LAB metabolites provides an opportunity to reduce or even inhibit a pathogen’s growth. Currently, antibiotic resistance among pathogenic bacteria is a real problem for humanity [17,18,19]. The increasing problem of drug-resistance among many pathogens, e.g., *Staphylococcus aureus*, *Pseudomonas aeruginosa, Klebsiella pneumonia* or *Escherichia coli,* requires the development of novel antiseptics [20,21,22,23]. One of the prospective alternatives to antibiotics is, as proposed in our study, the post-cultured synthesis of zinc oxide nanocomposites by lactic acid bacteria isolated from whey. The use of these natural raw materials as a source of a specific “bioreactor” capable of forming ZnO NCs perfectly fits in with the idea of environmental protection and sustainable development. Furthermore, using probiotic strains for nano-ZnO synthesis allows the formation of nanomaterials with specific organic deposits on their surfaces coming from microorganisms’ cells.

In our study, the probiotic strain (*Lactobacillus paracasei* LC20) isolated from sweet whey was chosen for the production of ZnO nanocomposites. Although whey is the subject of much controversy, because it can pose a risk to the environment if is not properly managed, it is, on the other hand, a rich source of valuable compounds (e.g., proteins, vitamins) and lactic acid bacteria (LAB) [24,25]. Due to the rich composition of whey, it is very important to find new, easy and environmental friendly technology not only for the utilization of this product but also for using it as a precious raw material.

Therefore, the main aim of this work was to carry out post-cultured ZnO nanocomposite synthesis by *Lactobacillus paracasei* LC20 isolated from sweet whey. The use of whey in the role of natural raw material creates new possibilities to apply it as a novel and eco-friendly source of LAB strains and, consequently, for the nano-ZnO. The obtained ZnO NCs were physicochemically characterized by different instrumental techniques. Based on the spectroscopic (FT-IR) and spectrometric (LDI-MS, spectrofluorimetry) experimental data, the presence of specific organic deposits on the ZnO NCs’ surfaces, together with the improvement of the fluorescence in the presence of ZnO NCs, was demonstrated. The antibacterial properties of biologically synthesized nanocomposites were investigated against both Gram(+) and Gram(−) drug-resistant bacteria strains by determining the MIC value (86.25–172.5 μg/mL) which indicates the antiseptic ZnO NC activity among relevant pathogens. The described unique attributes (organic surface deposits or luminescence properties) of bio-synthesized ZnO nanocomposites indicate the potential for their further application in both medicine and engineering fields. Finally, the results of MS analysis also support the previous description of ZnO NCs’ formation mechanism.

## 2. Materials and Methods

### 2.1. Isolation and Identification of Bacterial Strain

The probiotic strain was isolated from sweet whey at pH > 6 (Dairy Cooperative in Drzycim, Poland) according to the previous protocol [26]. The molecular identification of bacterial strain was performed by MALDI-TOF-MS identification by the ultrafleXtreme mass spectrometer (Bruker Daltonics, Hamburg, Germany) using formic acid-acetonitrile extraction and BioTyper identification [26]. Finally, the isolated strains of *L. paracasei* LPC20 were deposited in the Polish Collection of Microorganisms (PCM) under deposit no. B/00287.

### 2.2. Extracellular Synthesis of Zinc Oxide Nanocomposites (ZnO NCs)

For the extracellular synthesis of ZnO NCs, the *Lactobacillus paracasei* LPC20, from the collection of Centre for Modern Interdisciplinary Technologies, Nicolaus Copernicus University in Toruń, isolated from sweet whey (Dairy Cooperative in Drzycim, Drzycim, Poland) [27], was chosen. The *L. paracasei* LPC20 strain was grown on Müller–Hinton broth (MH, Sigma-Aldrich, St. Louis, MO, USA) and incubated at 37 °C for 24 h. After the incubation, the culture was centrifuged at 12,000 rpm for 30 min and the cell-free supernatant was collected and used for further ZnO NC synthesis. As a precursor of ZnO, zinc nitrate (Zn(NO_3_)_2_) at 0.1 g/mL concentration was chosen and added to the supernatant under continuous magnetic stirring. Then, the synthesis was carried out at 60 °C for 1 h. After this time, the mixture was centrifuged (7000 rpm, 15 min) and the obtained supernatant was transferred to a new container and heated at 100 °C until a dried precipitate appeared. The collected ZnO powder was washed with distilled water three times. Then, unbounded ions as well as low molecular weight metabolites were removed by 5-day dialysis (3 kDa cutoff, Spectrum Lab, Thermo Fisher Scientific, Vantaa, Finland). As a control, MH broth instead of *L. paracasei* LCP20 supernatant was used.

### 2.3. Physicochemical Characterization of ZnO NCs

#### 2.3.1. Zeta Potential Measurement

The ZnO NC zeta potential value was determined with Zetasizer Nano Series (Malvern Instruments, Malvern, Great Britain). Before measurement, the ZnO NCs (at 0.05 mg/mL concentration) were suspended in water at specific pH (range of 2 to 11) and mixed for 5 min. The measurements included three repetitions for each sample. The zeta potential data obtained were processed using Curve Expert Professional 2.6.5 software (Hyams Development, Huntsville, AL, USA). According to the electrokinetic theory, the processed data were fitted to the rational model. All measurements were carried out in triplicate.

#### 2.3.2. Fourier Transform Infrared Spectroscopy Analysis

Concerning the active functional groups present on ZnO NCs, FT-IR analysis was performed using a Spectrum 2000 (Perkin-Elmer, Waltham, MA, USA) in the range of υ = 400–4000 cm^−1^. The sample was prepared by application of the KBr disc method. The spectroscopic data were processed with OPUS 7.5 software (Bruker Daltonics, Hamburg, Germany).

#### 2.3.3. Transmission Electron Microscopy (TEM) and Energy Dispersive X-ray (EDX) Analysis

The structure and surface morphology of the obtained ZnO NCs were investigated by scanning electron microscopy with focused ion beam (SEM/FIB—Quanta 3D FEG, FEI, Gräfelfing, Germany). The confirmation of ZnO nanoparticles’ presence in nanoscale was examined by transmission electron microscopy (TEM, FEI Tecnai F20 X-Twin, Hillsboro, OR, USA) coupled with energy dispersive X-ray (EDX) detector (RTEM SN9577, 134 eV, Edax, Mahwah, NJ, USA). For SEM analysis, ZnO NC powder was used, whereas for transmission electron microscopy investigation, sample solution was put on a carbon-coated grid and the excess solution was removed.

#### 2.3.4. Spectrofluorometric Analysis

The ZnO NCs were analyzed with a JASCO FP-8300 spectrofluorometer (JASCO Europe, Cremella, Italy) and the three-dimensional (3D) excitations and emission spectra were recorded with 5 nm wavelength interval in the 210–735 nm range. As a control sample, zinc nitrate and water were used.

#### 2.3.5. X-ray Diffraction Study

In order to characterize the crystalline structure of the obtained ZnO NCs, the X’Pert Pro Analytical diffractometer (Phillips, Erlangen, Germany) equipped with CuKα (λ = 1.54056 Å) radiation source and Ni filter was used. The powder sample was analyzed and the crystallite size of sample was calculated based on the Scherrer equation [28]. The registered XRD pattern was processed using XRD Malvern Panalytical software (version 1.5a (1.5.1.135), Almelo, The Netherlands). As a control sample, MH broth instead of *L. paracasei* LCP20 supernatant as well as the chemically obtained ZnO NCs were used.

#### 2.3.6. Thermogravimetric Analysis (TG-DTA)

Thermal analysis of the obtained nanomaterial was performed by using TA Instruments type SDT 2960 (Artisan Technology, Champaign, IL, USA) in the range of 0–1000 °C with an air flow rate of 100 mL/min and heating rate of 10 °C/min. All data were processed by TGA-DTA thermal analysis software (version V5 7.0, TA Instruments, New Castle, DE, USA). All measurements were carried out in triplicate.

#### 2.3.7. Laser Desorption/Ionization with Mass Spectrometry Analysis (LDI-TOF-MS)

The LDI-TOF-MS analysis was performed using ultrafleXtreme mass spectrometer (Bruker Daltonics, Hamburg, Germany) and ZnO NCs suspended in water were spotted on the ground steel target (Bruker Daltonik, Bremen, Germany) according to the previously described protocol [10] with the required modification (without the α-Cyano-4-hydroxycinnamic acid (HCCA) matrix). Protein Calibration Standards I (Bruker Daltoniks, Bremen, Germany), Peptide Calibration Standard and two signals characteristic for the matrix [M-H]^+^ and [2M-H]^+^ were used for the external calibration, according to the standardized Bruker sample preparation procedure. Molecular fingerprint (MF) spectra of ZnO NCs were recorded in reflectron positive mode, within a *m*/*z* range of 100–3500, and we applied an acceleration voltage of 25 kV. Fragment spectra were recorded using the LIFT default method (Bruker Daltonics, Hamburg, Germany) at 100% of laser power, global attenuator 50% with calibration on the immonium ions [29,30]. The voltage on the LIFT electrodes was 19.0 and 2.7 kV, respectively. MS spectra were registered in FlexControl (Bruker Daltonics, Hamburg, Germany), while the FlexAnalysis (Bruker Daltonics, Hamburg, Germany) was used for data analysis.

### 2.4. Antimicrobial Potential of ZnO NCs

#### Colony Forming Unit (CFU) and Minimal Inhibitory Concentration (MIC)

Four different relevant microorganisms such as *Staphylococcus aureus* ATCC 11632, *Pseudomonas aeruginosa* ATCC 15441, *Klebsiella pneumoniae* ATCC BAA-1144 and *Escherichia coli* ATCC 25922, received from the Pol-Aura company (Pol-Aura, Olsztyn, Poland), were chosen for the antimicrobial activity of extracellularly synthesized ZnO NC determination. All chosen bacterial strains were revived in Mueller–Hinton (MH) broth to 1 × 10^6^ CFU/mL. The concentration of ZnO NCs was determined by inductively coupled plasma mass spectrometry (ICP-MS) analysis and dilutions of ZnO NCs were prepared (172.5, 86.25, 43.125, 21.56, 10.78, 5.39, 2.69 and 1.35 μg/mL, respectively). The growth bacterial cells (1 × 10^6^ CFU/mL) were mixed with the prepared concentration in the ratio 1:1. To the prepared mixture, 12 μL of in vitro toxicology assay kit, resazurin based (Sigma-Aldrich, St. Louis, MO, USA) was added. After 24 h of incubation at 37 °C, the MIC value was determined visually based on the change in the redox indicator color from blue to pink or colorless. The lowest concentration at which no change in color was observed was considered as the MIC value, according to [31]. All the experiments were prepared in triplicate.

Additionally, all the bacteria were subjected to the colony forming unit (CFU) assay. To determine the sensitivity of bacterial strains to biologically synthesized ZnO NCs, bacteria were grown in the presence of nanocomposites (at concentrations of 345, 172.5 and 86.25 μg/mL, respectively) at 37 °C for 24 h. After the incubation, various dilutions (10^−1^–10^−8^ folds) of bacterial suspension were prepared. Then, 100 μL of each sample was transferred to tryptic soy agar TSA plates, incubated at 37 °C for 24 h, and CFU were counted manually. NC-free bacteria strains were used as a negative control while ampicillin at 10 μg/mL concentration for *S. aureus, E.coli* and *K. pneumoniae*, and at 25 μg/mL concentration for *P. aureginosa*, was used as a positive control. The CFU assay was performed in triplicate. The concentration of ZnO NCs was estimated from the observed MIC values for each bacterial strain and the concentration of ampicillin was based on the European Committee on Antimicrobial Susceptibility Testing (EUCAST) breakpoints table.

## 3. Results and Discussion

The synthesis of ZnO nanomaterials can be carried out by using various types of biological sources such as proteins, plant extracts or microorganisms. Recent studies have demonstrated that both intra- and extracellular approaches are efficient enough to synthesize different types of nanoparticles, e.g., silver [10,27,32], gold [33] or titanium oxide [34] NPs; however, the microbial synthesis of nan-ZnO has still not been sufficiently described.

### 3.1. Physicochemical Characterization of ZnO NCs

**Figure 1:**
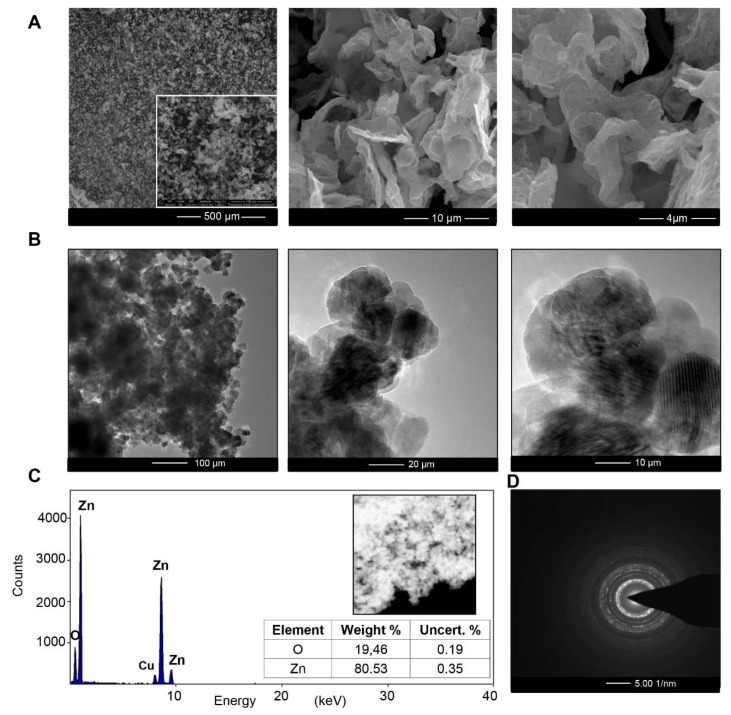
SEM micrograph (**A**), TEM micrograph (**B**), EDX spectra (**C**) and selected area (electron) diffraction (SAED) (**D**) of biologically synthesized ZnO NCs.

Figure 1A (SEM image) shows the homogenous structure of the biologically synthesized ZnO NCs. The TEM image of bio-ZnO NCs with the EDX spectra are shown in Figure 1B,C and indicate the presence of zinc (with approximately 1, 8.5 and 9.5 keV signals) and oxide as a major element in the sample [35]. Theoretically, the expected stoichiometric mass percent of oxygen and zinc is 19.7% and 80.3%, respectively. According to the EDX analysis (Figure 1C), the weight percentage of Zn and O in our sample was 19.46% and 80.53%, which is in with good correlation with the theoretical values [36]. Slight differences observed between the experimental and hypothetical data can be assigned to the presence of some organic deposits coming from the bacterial metabolites or proteins secreted by the bacterial strains during the inoculation step. Moreover, on the EDX spectra, the signal of copper is present—it corresponds to the TEM grid.

**Figure 2:**
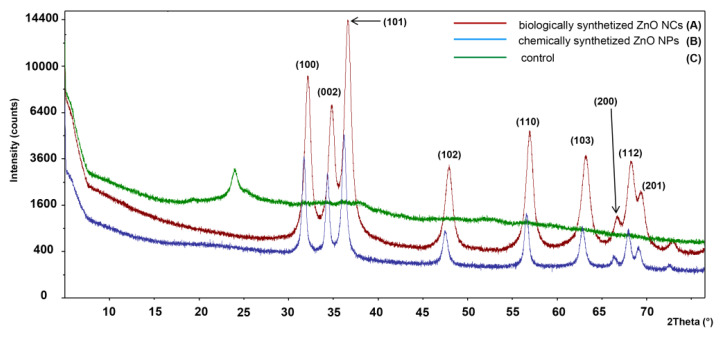
XRD pattern of biologically synthesized (**A**), reference ZnO NCs (**B**) and control (**C**).

The selected area of diffraction pattern (SAED) (Figure 1D) confirmed the highly crystalline nature of the synthesized nanoparticles, which is strongly related to the pattern obtained during XRD analysis (Figure 2). Typical ZnO peaks present at 2θ = 31.93°, 34.59°, 36.41°, 47.7°, 56.75°, 63.02° and 68.09° correspond to the Bragg reflections identified as (100), (002), (101), (102), (110), (103), (200), (112) and (201), respectively. Data from the XRD analysis clearly illustrate the formation of the wurtzite structure of zinc oxide (according to the Joint Committee on Powder Diffraction Standard (JCPDS) card no. 36-1451, P63mc) at the 13.70 ± 1.53 nm size (calculated from the Scherrer equation [28]). In Figure 2C, one additional peak around 25° was detected—it might be assigned to the zinc hydroxynitrate (JCPDS No: 72-0627). According to the literature data [37,38,39], zinc nitrate Zn(NO_3_)_2_ or its hydroxides is one of the intermediate products of ZnO NC synthesis. On the other hand, in the control sample (Figure 2C), no zinc oxide was formed, which indicates that only the specific combination of biologically active compounds from supernatant allows for effective ZnO NC formation. The difference between the ZnO NCs’ size estimated from the TEM and XRD analysis can be attributed to the fact that the particles are formed from more than one crystallite [40,41]. Furthermore, nanoparticles tend very often to create aggregates—therefore, the proper sample preparation steps are decisive [2]. In the case of the control sample, no zinc oxide was detected (Figure 2).

**Figure 3:**
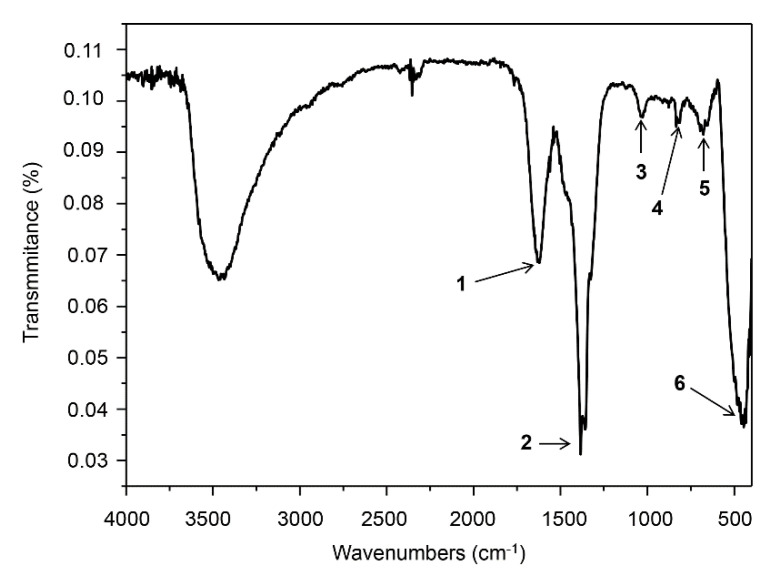
FT-IR spectra for biologically synthesized ZnO NCs in the υ = 400–4000  cm^−1^ range; υ (cm^−1^): 1: 1600–1700, 2: 1400, 3: 1100–1200, 4: 800–900, 5: 700–800, 6: 500–600.

**Figure 4:**
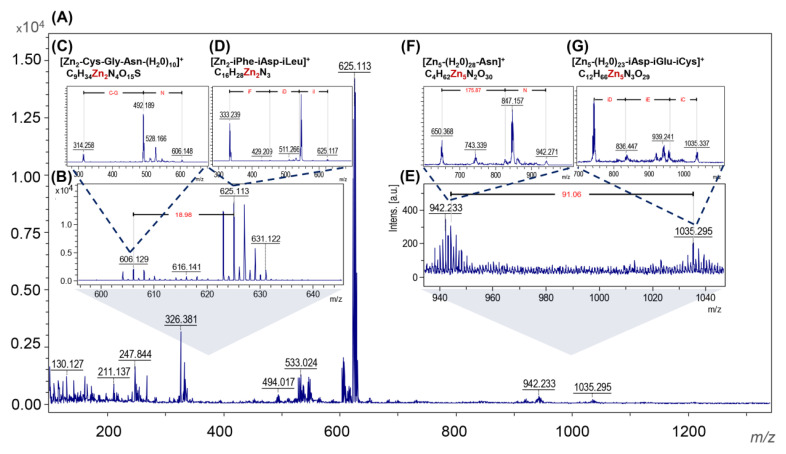
The molecular fingerprint of biologically synthesized ZnO NCs (**A**); the MS/MS spectra for specific signals (**B**–**G**).

The presence of organic deposits on the ZnO NCs’ surfaces was studied by both spectroscopic (FT-IR) (Figure 3) and spectrometric (LDI-MS) (Figure 4) techniques. Fourier-transform infrared spectroscopy proved the presence of functional groups which contributed to the process of nano-ZnO formation, while mass spectrometry is a useful technique for the analysis of metal/metal oxide nanomaterials with organic deposits, mainly due to the isotopic pattern of d-electron metals [42]. Figure 3 represents the biologically synthesized ZnO NC spectra in the mid-infrared (MIR) range. A signal at υ = 1587–1592 cm^−1^ (1) is associated with the NH_2_ scissoring vibration of phenylalanine (Phe) amino acid, whereas the vibration at υ = 1695 cm^−1^ is related to the Phe carboxylic acid (C = O stretching) [43,44]. The signal localized at υ =1648–1685 cm^−1^ (1) is often characteristic of leucine (Leu) δ_as_NH_3_^+^ side chain vibration [45,46]. Moreover, the change in the IR spectrum at 1560 cm^−1^ (1) is a result of the deprotonated carboxyl (COO^−^) group of glutamic and aspartic acids (Glu and Asp, respectively) [37,38,47]. In the ZnO NC FT-IR spectra, a signal at υ = 1400 cm^−1^ (2) was observed—it is correlated with the plane bending of methylene (−CH_2_) in the cysteine (Cys) or glycine amino acids [48,49]. In the range of 1100–1200 cm^−1^ (3), there is an area of C-C stretching: C-H and N-H bending vibrations are typical for amide III [44,46,50,51] but also for lactic acid and its metabolites [52]. Additionally, the signal at υ = 800–900 cm^−1^ (4) is related to the C-H vibrations of bioactive compounds included in the organic deposit on the ZnO NC surface (such as peptides or lipids) [10,47,52]. The band at υ = 730–770 cm^−1^ (5) corresponds to the aromatic amino acids side chains (e.g., in the phenylalanine) [43]. The FT-IR spectra (Figure 3) also confirmed the presence of zinc oxide formation—the peak at the region between 500 and 600 cm^−1^ (6) is related to the Zn-O bond [2,53].

**Figure 5:**
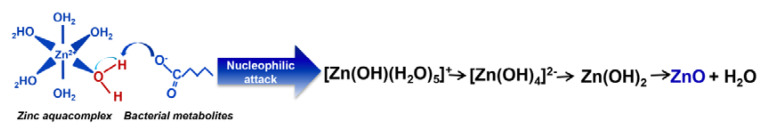
The proposed mechanism of ZnO NC formation.

The recorded molecular fingerprint of ZnO NC nanostructure-assisted laser desorption/ionization is shown in Figure 4A,B,E and presents the MS/MS analysis in LIFT mode of the 606.129, 625.113, 942.233 and 1035.295 *m*/*z* signals, respectively. The signal at 606.129 *m*/*z* (Figure 4C) was assigned to the ionized fragment of three amino acid (Cys, Gly and Asn) peptides with the bounds of 2 Zn^2+^ and 10 H_2_O molecules. Another is two Zn^2+^ bound to the fragments of three amino acids—phenylalanine, aspartic acid and isoleucine (625.113 *m/z*; Figure 4D). These two signals are linked with the water. LDI MS/MS measurements also showed the presence of a signal at 942.233 *m/z* [Zn_5_-(H_2_O)_28_-Asn]^+^ and 1035 *m/z* [Zn_5_-(H_2_O)_23_-iAsp-iGlu-iCys]^+^ signals (Figure 4F,G). What is interesting, between them, is that five molecules of water were found. The obtained results confirm the presence of organic deposits in the ZnO NC structure which is correlated with the FT-IR data. Moreover, the type of amino acid interacting with the zinc ions and the presence of water molecules is strongly associated with the previously described mechanism of ZnO NC formation [5,54]. The ability of metal ions to bind the specific protein functional groups is highly connected to Me^2+^ nature. Zinc, with its unique coordination chemistry, forms stable aqua complexes, [Zn(H_2_O)_6_]^2+^, which are able to exchange their water molecules when binding to other ligands, mainly nitrogen ones [55]. Many literature reports have demonstrated that Zn^2+^ bind preferentially to the proteins through negatively charged (aspartic and glutamic acids; Asp^−^ and Glu^−^, respectively) or polar residues (cysteine, histidine and asparagine) [56,57,58]. Molecular dynamics (MD) and density functional theory (DFT) calculations performed by our research group (not published yet) have indicated that the proton transfer reaction results in the formation of an aqua-hydroxo complex [Zn(OH)(H_2_O)_5_]^+^. Then, it can be transformed to zinc oxide, through [Zn(OH)_4_]^2−^ and Zn(OH)_2_. Therefore, the presented data confirmed the possible formation of Zn^2+^ (as an aqua complex) during biosynthesis and the mechanism of ZnO NC formation (Figure 5). To sum up, the MS/MS analysis confirm the data from the FTIR and point out the specific peptide fragments taking part in ZnO NC formation.

**Figure 6:**
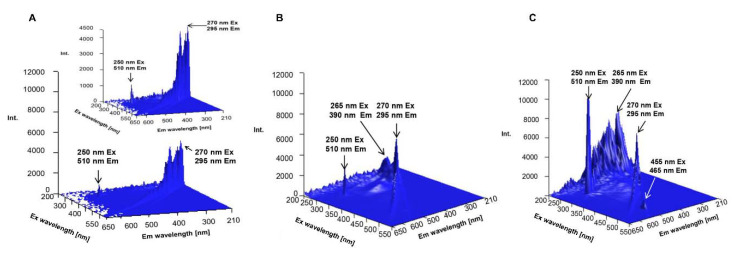
Fluorescence spectra of water (**A**), zinc nitrate (**B**), biologically synthesized ZnO NCs (**C**).

As a complementary analysis, the 3D spectrofluorometric profiles of biologically synthesized ZnO NCs, water and zinc nitrate (as a control) were recorded (Figure 6). In the case of the obtained nanocomposites (Figure 6C), the excitation and emission wavelengths were found to be 270, 295 nm (I); 250, 510 nm (II), 455, 465 nm (III) and 265, 390 nm (IV), respectively. Band no. IV is characteristic of the zinc oxide optical band gap [11,54,59] while band no. III might be correlated with the presence of organic deposits in the ZnO NC structure. In comparison to the control samples, the presence of the biologically synthesized nano-ZnO generated an increase in quartz fluorescence intensity (band II)—it is strongly related with the surface plasmon resonance (SPR) effect [60]. Surface plasmon resonance occurs as a resonance effect due to the interaction of nanoparticle conduction electrons with incident photons [61]. This interaction is related to a large enhancement of the field intensity and, consequently, with the enhancement of the fluorescence signal [60,62,63]. A similar effect was presented by many researchers [5,10,54,62] as well as in our previous work [54]—the addition of synthesized ZnO NCs enabled an improvement in the intensity of fluorescence emission. The consequences of the observed surface plasmon resonance, such as the increase in the fluorescence signal, create new application possibilities for biologically synthesized ZnO NCs, e.g., as biosensors in medical diagnostics. Currently, analytical chemistry is based, more and more, on modern instruments, and scientists have at their disposal a wide range of different analytical methods. Almost every week, new papers, works and the development of new approaches are presented. Many of them are used not only in the chemistry field but also in biological and nanotechnology sciences or in medicine as well. Among others, NALDI-TOF/MS (nonporous-assisted laser desorption/ionization time-of flight mass spectrometry) is arousing a lot of interest. It is a matrix-free soft laser base MS technology mainly used for low molecular weight biomolecules and organic molecules and classified as the new generation of mass spectrometry [64,65]. Previous studies have indicated that an inorganic matrix with large surface area, high photon absorption efficiency and low heat capacity is suitable for realizing effective desorption and ionization. According to this information, nano-ZnO, as a semi-conductive material, can absorb energy from the laser in wavelengths typically used in MALDI and transfer it to the analyte [64]. Watanabe et al. [66] have used ZnO nanoparticles for the desorption/ionization of several small molecules, which is still an analytical challenge. Kang et al. [64] compared a suspension of analyte/ZnO nanowires and the direct deposition of the analyte onto the surface of a nanowire chip. Taking this into consideration, the results of our study have shown that biologically synthesized ZnO NCs seem to be a promising matrix for LDI experiments since, in this work, the MALDI analysis was performed without any matrix.

**Figure 7:**
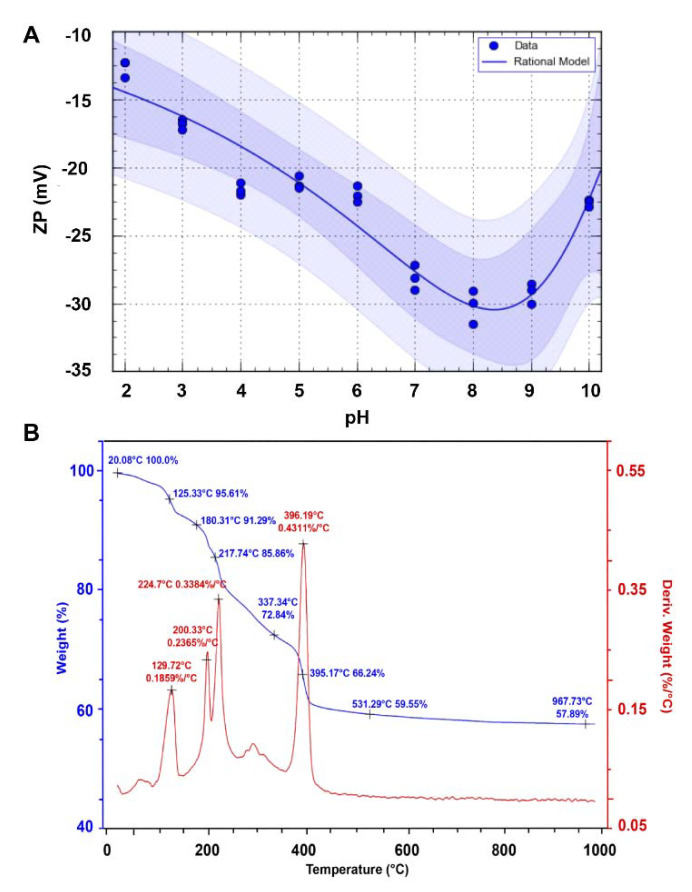
Zeta potential in the 2–10 pH range (**A**) and thermogravimetric TG/DTG curves (**B**) for biologically synthesized ZnO NCs.

The stability of the obtained nanomaterials was estimated by the zeta potential measurement. According to the electrokinetic theory and Smoluchowski equation, biocolloids have a surface charge that attracts a thin layer of ions of the opposite charge to the nanoparticle surface. Consequently, the electric potential at the boundary of the double layer is called the zeta potential of the NCs. In our study, in a range of pH 2–6, the biologically synthesized ZnO NCs are unstable and might create some aggregates—their zeta potential value varied from −12.63 ± 0.66 to −22.01 ± 0.59 mV (Figure 7A). Instability of the system in this pH scope is associated with the deprotonation of organic deposits (such as carboxyl groups on the NC’s surface) [32]. Above pH 6, the zeta potential (ZP) value slightly decreased, and at pH 7–9, the average ZP value at −29.15 ± 1.05 mV clearly indicated that ZnO nanocomposites are fairly stable. Taking into consideration the literature data, the ZP value of NCs formed in our study is higher than the zeta potential of ZnO NPs synthesized intracellularly with *L. plantarum* (−15.3 mV) [14], *L. paracasei* LB3 (−16.2 mV) [54], as well as those obtained during extracellular synthesis by using *R. pyridinivorans* (−15.5 mV) [11]. Above pH 9, the ZP value was higher again (−22.57 ± 1.15 mV) and the system was unstable. This phenomenon can be interpreted as the transformation of colloidal Zn(OH)_2(S)_ to Zn(OH)_2(aq)_ ions in the alkaline pH region [67]. In neutral aqueous conditions, the Zn^2+^_(aq)_ and Zn(OH)^+^_(aq)_ ions are in equilibrium with surface hydroxide, while at higher pH, the dominant species are Zn(OH)_2(aq)_, which are able to precipitate and create aggregates. In consequence, the stability of nano-ZnO is definitely lower [67,68,69].

Thermal analysis of the nanomaterials allowed us to indicate their stability according to how they change depending on the temperature. In Figure 7B, the TG and DTG curve for the ZnO NCs synthesized by the extracellular approach is presented. As can be observed, the slope of the TG curve is initially constant and progresses horizontally, which demonstrates that the obtained nanomaterial is pure and does not contain any impurities. Data form thermal analysis indicate good thermal stability up to 130 °C—at this temperature, the powder starts to decompose in a rapid succession of reactions. According to Figure 6B, four different weight loss steps occurred. The initial weight loss at 129.72 °C, with the weight loss rate at 0.1859%/°C, is related to the removal of residual water. The second and third stages are similar to each other (200.33 °C and 224.7 °C, respectively) and appeared due to the elimination of coordinated water molecules [70,71]. The biggest change in the weight loss was observed from 217.74 °C (85.86% of weight) to the 396.19 °C (66.24% of weight)—it might be assigned to the decomposition step, including the removal of the organic deposit of nanocomposites and ending with the formation of ZnO [70,71,72]. No weight decline was observed at temperatures above 500 °C. Vasile et al. [40] showed the good thermal stability of chemically synthesized ZnO nanocomposites to 200 °C, whereas Basha et al. [73] described the green synthesis of nano-ZnO by using an alginate with thermal stability up to 160 °C. Based on these data, it can be concluded that biosynthesis allowed the formation of slightly less thermostable ZnO NCs, which is closely related to values reached by our group.

### 3.2. Antimicrobial Activity of ZnO NCs

**Figure 8:**
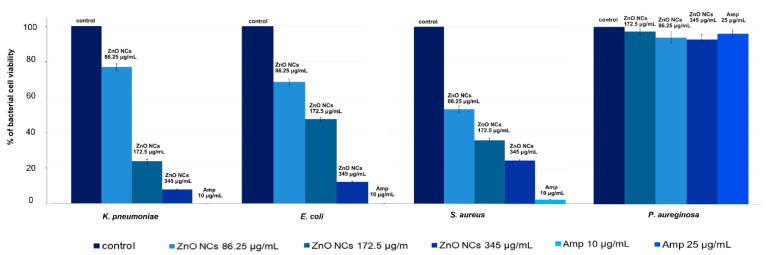
Quantification of bacterial cell viability (%) at different concentrations of ZnO NCs and ampicillin.

As shown in Table 1, the minimal inhibitory concentrations of biologically synthesized ZnO NCs were in the range of 86.25–172.5 μg/mL. The maximum MIC value was found to be 172.5 μg/mL for the *E. coli* ATCC 25922 and *K. pneumonia* ATCC BAA-1144 strains. On the other hand, *S. aureus* ATCC 11632 was susceptible to ZnO NC treatment at the lowest concentration (86.25 μg/mL). The obtained nano-ZnO in our study did not present antibacterial effects against *P. aureginosa* ATCC 15441 (Table 1). Furthermore, the experimental MIC values were in agreement with the CFU/mL values for each strain treated with nanocomposites. For the control sample of *K. pneumonia*, *E. coli*, *P. aeruginosa* and *S. aureus*, the CFU/mL values were 3.13 × 10^9^, 2.81 × 10^9^, 3.32 × 10^9^ and 3.32 × 10^9^, respectively. The ZnO NCs at 172.5 μg/mL were sufficient to inhibit the growth of *K. pneumonia* (7.41 × 10^8^ CFU/mL) and *E. coli* (1.28 × 10^9^). The MIC value for the *S. aureus* was found to be 86.25 μg/mL and this concentration of nanocomposites reduced bacterial cells to 1.79 × 10^9^ CFU/mL. The *P. aureginosa* strain was resistant to the ZnO NC treatment and the average CFU value was at the 3.16 × 10^9^ level. As a positive control, ampicillin was applied—all bacterial strains except *P. aureginosa* were found to be sensitive to antibiotic treatment. Ampicillin at 10 μg/mL concentration reduced the *K. pneumonia*, *E. coli* and *S. aureus* cell numbers to 2.30 × 10^9^, 1.67 × 10^5^ and 8.27 × 10^7^, respectively. Colony forming units were also quantified for all bacterial cells and represented as percentage of viable cells in comparison to control sample (untreated culture) (Figure 8).

In the present study, antibacterial properties of the ZnO NCs are slightly different to those described in previous reports related to nano-ZnO biological synthesis. According to [74], ZnO NPs produced by cyanobacterium *Nostoc* sp. [7] showed MIC values for *S. aureus* and *E. coli* of 64 μg/mL and 2000 μg/mL, respectively. Jayaseelan et al. [75] tested the intracellularly synthesized nano–ZnO by *A. hydrophila* against a few bacterial strains and they obtained the MIC in the 1.2–2.9 μg/mL range. In our study, for *S. aureus*, the lowest concentration was 86.25 μg/mL, whereas for the *E. coli* and *K. pneumoniae* strains, the concentration of ZnO NCs at 172.5 μg/mL was enough to inhibit bacterial growth. Both *S. aureus* and *E. coli* have become a serious problem progressively observed not only in humans but also in veterinary medicine worldwide [22,76]. Although these bacteria strains are part of the commensal flora, they can also become opportunistic pathogens [77,78]. For instance, *Staphylococcus aureus* normally colonizes the nasal cavity. After breaking through its normal habitat, *S. aureus* is able to cause a number of infections, mainly skin or bloodstream infections and pneumonia. It has been widely observed that *S. aureus* has developed extraordinary resistance to almost all classes of antibiotics [21,23]. One of the bacterial strategies of protection from drugs and antibiotics is biofilm formation [79]. Strains tested in our study, such as *S. aureus* and *P. aureginosa*, have the propensity to form biofilms and then the proper antibiotic treatment is much more difficult [80]. One approach to controlling the production of these might be nanomaterials with antibacterial capabilities [81,82]. Some scientific reports have shown that nanoparticles have been effectively applied to eliminate preformed biofilms or to stop their formation on the surfaces of medical equipment [83,84]. Eshed et al. [85] reported that ZnO NPs presented significant reduction in biofilm formation by *Streptococcus* mutants. Another work [86] indicated the ability of nano-ZnO to inhibit biofilms formed by vancomycin-resistant *S. aureus.* Therefore, the antibacterial activity of synthesized ZnO NCs in our study might be associated with their anti-biofilm behavior. However, our data also pointed out the absence of antibacterial activity against *P. aeruginosa* ATCC—this strain is able to form a biofilm which protects the pathogen from host immune response and antimicrobial therapy [87]. Compared with *Staphylococcus aureus*, *P. aeruginosa* is a Gram(−) bacteria. Consequently, due to the various cell wall structures, the antibacterial activity of zinc oxide NCs is significantly different against Gram(+) and Gram(−) strains. The cell wall of Gram(−) species consist of lipopolysaccharides (LPS), lipoproteins and phospholipids, which act as a penetration barrier for nanostructures [88,89]. Taking this into consideration, it can be concluded that the entry of ZnO NCs through the *P. aureginosa* cell wall is more difficult and thus they possess every antibacterial effect.

Another example of the opportunistic pathogens causing a broad spectrum of diseases and showing increasingly frequent acquisition of resistance to antibiotics is *Klebsiella pneumoniae* [20]. Hameed et al. [90] tested chemically synthesized ZnO NPs doped with Nd against *K. pneumoniae*—the minimal inhibitory concentration was found to be 1000 μg/mL. The work of Ansari et al. [91] pointed out that the minimum concentration of ZnO nanoparticles synthesized by the sol-gel method at 100 μg/mL inhibited the colony forming ability of *K. pneumoniae*. In comparison with the literature data, the MIC values for *K. pneumoniae* were found to be much lower. Therefore, it cannot be excluded the fact that the higher antibacterial effects of biologically synthesized ZnO nanoparticles using *L. paracasei* LPC20 in the present study are due to the existence of the organic deposit on their surface.

## 4. Conclusions

This study presents, for the first time, the use of *Lactobacillus paracasei* LPC20 strain isolated from whey as a new and efficient source for post-cultured ZnO nanocomposite synthesis. An interdisciplinary approach, including a wide range of instrumental techniques, was used to evaluate the structure, size, stability and organic deposits of the obtained nanocomposites. Scanning electron microscopy revealed the surface morphology and structure of biologically obtained nano-ZnO, while the transmission electron microscopy and diffraction study pointed out the formation of nanocomposites and confirmed the chemical composition of the sample. Biologically synthesized ZnO NCs exhibit dispersion stability in the 7–9 pH range, with the average ZP value at the −29.15 ± 1.05 mV level and thermal stability up to 130 °C. Results of spectroscopic (photoluminescence and FT-IR) and spectrometric analysis pointed out the presence of organic deposits on the ZnO NCs’ surfaces. According to the FT-IR data, the presence of signals corresponding to the deprotonated carboxyl groups of amino acids, mainly glutamic and aspartic acids, indicated their crucial participation in the biosynthesis process. Moreover, the FT-IR spectra also confirmed the presence of zinc oxide—the peak at the region between 500 and 600 cm^−1^ was related to the Zn-O bond, which was in good agreement with the TEM/EDX and XRD data. The LDI-TOF MS/MS approach allowed identification of the amino acid sequences naturally present on the surfaces of ZnO NCs and an understanding of which types of amino acids might be linked to or interact with Zn^2+^. The outcomes of our investigation additionally give an insight into our previous study and allow a clearer understanding of the mechanism of ZnO NC formation. Zinc is present in the form of an aqua complex and as an electron acceptor can interact with deprotonated carboxyl groups of bacterial enzymes or released metabolites. Based on the LDI-MS data and our previous work, the main role of amino acid functional groups (such as COO^−^) in the subsequent aqua-hydroxo complex formation is strongly confirmed. Then, through further reaction, [Zn(OH)_4_]_2_ is transformed into ZnO. Furthermore, the fluorescence of biologically synthesized nanocomposites and the amplification of the signal in the presence of ZnO NCs, as well as the LDI analysis, create new application possibilities, e.g., as biosensors or matrixes in mass spectrometry.

Additionally, the biologically synthesized ZnO NCs exhibited good antimicrobial properties against clinically relevant *Escherichia coli* ATCC 25922, *Staphylococcus aureus* ATCC 11632 and *Klebsiella pneumoniae* ATCC BAA-1144. The MIC values obtained in our study are comparable to or even lower than those reported in the literature. Furthermore, the experimental MIC values were confirmed by the complementary colony forming units (CFU) assay. Accordingly, biologically synthesized ZnO NCs might be potentially used as a novel, easy-to-handle and antiseptic agent for external use, e.g., in bedsore treatment as a new type of skin path or in ointment formulations. Nevertheless, a further study regarding antibacterial activity against an expanded range of species by using other complementary techniques as well as cytotoxicity tests of ZnO NCs is needed.

## Figures and Tables

**Table 1:** Minimum inhibitory concentration (MIC) of the biologically synthesized ZnO NCs against tested microorganisms.

Tested Material	*Klebsiella Pneumoniae*	*Escherichia Coli*	*Pseudomonas Aeruginosa*	*Staphylococcus Aureus*
Access no.	ATCC BAA-1144	ATCC 25922	ATCC 15441	ATCC 11632
*ZnO NCs*	172.5 μg/mL	172.5 μg/mL	-	86.25 μg/mL
